# Soy Extract, Rich in Hydroxylated Isoflavones, Exhibits Antidiabetic Properties In Vitro and in *Drosophila melanogaster* In Vivo

**DOI:** 10.3390/nu15061392

**Published:** 2023-03-14

**Authors:** Kai Lüersen, Alexandra Fischer, Ilka Bauer, Patricia Huebbe, Yukiko Uekaji, Keita Chikamoto, Daisuke Nakata, Naoto Hiramatsu, Keiji Terao, Gerald Rimbach

**Affiliations:** 1 Institute of Human Nutrition and Food Science, University of Kiel, 24118 Kiel, Germany; 2 CycloChem Bio Co., Ltd., 7-4-5 Minatojima-Minamimachi, Chuo-ku, Kobe 650-0047, Hyogo, Japan; 3 Toyo Hakko Co., Ltd., 1-39-1 Yoshikawa-cho, Obu-shi 474-0046, Aichi, Japan

**Keywords:** soy, isoflavones, hydroxy isoflavones, bioactivity, glucose metabolism

## Abstract

In the context of the growing prevalence of type 2 diabetes (T2DM), control of postprandial hyperglycemia is crucial for its prevention. Blood glucose levels are determined by various factors including carbohydrate hydrolyzing enzymes, the incretin system and glucose transporters. Furthermore, inflammatory markers are recognized predictors of diabetes outcome. Although there is some evidence that isoflavones may exhibit anti-diabetic properties, little is known about to what extent their corresponding hydroxylated metabolites may affect glucose metabolism. We evaluated the ability of a soy extract before (pre-) and after (post-) fermentation to counteract hyperglycemia in vitro and in *Drosophila melanogaster* in vivo. Fermentation with *Aspergillus* sp. *JCM22299* led to an enrichment of hydroxy-isoflavones (HI), including 8-hydroxygenistein, 8-hydroxyglycitein and 8-hydroxydaidzein, accompanied by an enhanced free radical scavenging activity. This HI-rich extract demonstrated inhibitory activity towards α-glucosidase and a reduction of dipeptidyl peptidase-4 enzyme activity. Both the pre- and post-fermented extracts significantly inhibited the glucose transport via sodium-dependent glucose transporter 1. Furthermore, the soy extracts reduced c-reactive protein mRNA and secreted protein levels in interleukin-stimulated Hep B3 cells. Finally, supplementation of a high-starch *D. melanogaster* diet with post-fermented HI-rich extract decreased the triacylglyceride content of female fruit flies, confirming its anti-diabetic properties in an in vivo model.

## 1. Introduction

The prevalence of diabetes, especially type 2 diabetes mellitus (T2DM), is increasing globally [1]. This metabolic disease is characterized by hyperglycaemia, induced by a progressive insulin secretory defect or a diminished or missing response of insulin receptors [2]. Continuously high blood sugar levels may result in long-term complications, including renal and cardiovascular diseases, retinopathy or an impaired blood flow [3]. Thus, controlling postprandial hyperglycemia through dietary means is crucial for the prevention of T2DM. Blood glucose levels are determined by various factors including food constituents and food matrix, glucose transporters, carbohydrate hydrolyzing enzymes (e.g., α-glucosidase, α-amylase) and hormones such as the incretin system. The incretin system relates to the gut hormones glucagon-like peptide-1 (GLP-1) and glucose-dependent insulin polypeptide (GIP), which increase postprandial insulin production by acting on pancreatic beta cells. Dipeptidyl peptidase-4 (DPP-4) degrades circulating GLP-1 and GIP and reduces the circulating postprandial glucagon level [4]; hence, DPP4 inhibitors are regarded as a novel means for extending the action of insulin and treating T2DM. In addition, intestinal glucose absorption is largely achieved by sodium/glucose symporter 1 (SGLT1) [5]. SGLT1 expression is regulated by the diet, e.g., it is strongly elevated in response to intraluminal glucose and by compounds that activate sweet taste receptors [6]. Moreover, it is induced in patients with T2DM [7]. The development of T2DM is usually associated with chronic inflammation. Accordingly, C-reactive protein (CRP), which is considered a sensitive systemic marker of low-grade inflammation, has been found to be a proper biomarker for T2DM [8]. CRP predominantly synthesized and secreted by hepatocytes is elicited by the dual activity of interleukin (IL)-6 and IL-1ß, and enhances inflammatory pathways by inducing IL-6 secretion [9,10]. A recent meta-analysis investigated the impact of soy intake on inflammatory markers and revealed significantly decreased CRP levels in women; however, the underlying mechanisms by which soy foods and their ingredients influence inflammatory biomarkers has not yet been elucidated [11].

Importantly, legumes, specifically soybeans, are a widespread dietary source of isoflavones, with genistein, daidzein and glycitein forming the major fraction [12]. In contrast, their hydroxylated counterparts, such as 8-hydroxygenistein (8OHGen), 8-hydroxydaidzein (8OHDai) and 8-hydroxyglycitein (8OHGly), are barely found in plants. Hydroxylation at either the C6 or C8 carbon position of the isoflavone backbone is not prioritized during isoflavone biosynthesis in plants, as cytochrome P450 (CYP)-dependent enzymes from plants seem to not catalyze the ortho-hydroxylation. However, food processing can affect the isoflavone concentration and isomer composition of soy products. Thus, most hydroxylated isoflavones (HI) are derived from fermented soybean foods, such as miso, natto, soy sauce and tempeh, where microorganisms, mainly fungi (e.g., *Aspergillus*) and bacteria (e.g., *Rhizopus, Streptomyces*), incorporate the hydroxyl-group into the isoflavone molecule during fermentation in a CYP-dependent manner [12,13]. Furthermore, the production of HIs is also feasible via microbial production using genetic engineering [14]. In addition, fermentation increases the cleavage of glycoside bonds of isoflavones, thereby enhancing their bioavailability [15,16].

**Figure 1:**
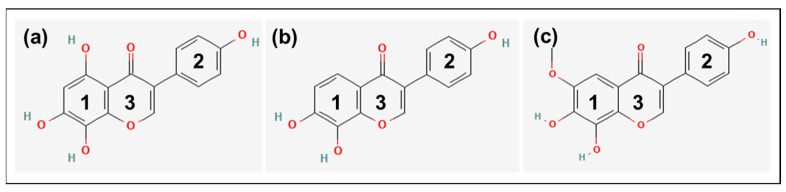
Chemical structure of (**a**) 8-hydroxygenistein (8OHGen; 4′,5,7,8-Tetrahydroxyisoflavone; C_15_H_10_O_6_, PubChem CID: 5492944), (**b**) 8-hydroxydaidzein (8OHDai; 7,8,4′-Trihydroxyisoflavone; C_15_H_10_O_5_, PubChem CID: 5466139) and (**c**) 8-hydroxyglycitein (8OHGly; 7,8,4′-Trihydroxy-6-methoxyisoflavone; C_16_H_12_O_6_, PubChem CID: 10870296). Structures were taken from PubChem [17]. **1:** Benzene ring 1; **2:** Benzene ring 2; **3:** Heterocyclic pyrone ring.

The basic chemical structure of the isoflavones consists of two benzene rings (1 and 2) linked via a heterocyclic pyrone ring (3) (Figure 1).

Isoflavones have been shown to induce endogenous antioxidant defense mechanisms, such as glutathione peroxidase, catalase and superoxide dismutase [18], presumably via an Nrf2-dependent signal transduction pathway [19]. Furthermore, an inhibition of lipoxygenase due to isoflavones has been described [20]. In addition, the dietary intake of genistein and daidzein improved the resistance of LDL against oxidation [21].

Genistein and daidzein are relatively weak scavengers of hydroxyl, superoxide, and nitric oxide free radicals, as determined using spin trapping and electron spin resonance spectroscopy [22]. Interestingly, it has been shown in vitro that the corresponding hydroxylated metabolites of genistein and daidzein exhibited significantly stronger bioactivity in terms of prevention of lipid peroxidation as compared to the parent compounds per se [23,24]. Hirota et al. [25] found that, among various isoflavones isolated from soybean miso, 8OHGen represented the most potent antimutagenic and antiproliferative activity. Likewise, in contrast to daidzein, 8OHDai has been found to be a potent aldose reductase inhibitor in vitro [26] and may, therefore, represent a potential substance for the treatment of diabetic complications.

However, fermented isoflavones remain an “understudied” group of soy compounds and little is known about the bioactivity of isoflavones regarding their antidiabetic properties [15], especially to what extent their corresponding hydroxylated metabolites, including 8OHGen, 8OHDai and 8OHGly, may affect glucose metabolism and biomarkers of inflammation. The aim of our study was to investigate a soybean extract before (pre-) fermentation and, in particular, a HI-rich extract after (post-) fermentation, regarding their antidiabetic and anti-inflammatory properties in vitro. *Aspergillus* sp. JCM 22,299 was applied for fermentation, which resulted in a substantial increase in HI, where 8OHGen represented the highest fraction. We tested the in vitro impact of the HI extract on α-glucosidase inhibition, starch digestion via α-amylase and the incretin system regarding DPP4 inhibition. The influence of soy bean pre-extract and HI extract was further evaluated based on the activity of glucose transport by SGLT1 using the Caco-2 cell culture model and CRP expression (mRNA and secreted protein level) in cultured hepatocytes.

In order to take into account pharmacological aspects, such as bioavailability, the influence of the gut microbiota and biotransformation, the verification of in vitro bioactivities in animal models usually represents the next important step in the development of new therapeutic approaches. In recent years, the fruit fly *Drosophila melanogaster* has been acknowledged as a valuable model in food science [27]. In particular, owing to the remarkable similarity between *D. melanogaster* and humans in terms of the metabolic pathways involved in energy metabolism and its hormonal regulation, several genetic and diet-based diabetes models have been established in fruit flies [28,29,30], which can serve as helpful tools to test putative antidiabetic substances [31,32]. Accordingly, we employed a high-sugar diet *D. melanogaster* obesity model, which we have previously used to validate the efficacy of plant extracts with α-amylase and α-glucosidase inhibitor activities [32] to examine whether the post-fermented HI extract exerted antidiabetic properties in vivo.

## 2. Materials and Methods

### 2.1. Preparation of Pre-Fermented and Hydroxy-Isoflavone (HI)-Enriched Post-Fermented Soybean Extract and Isoflavone Analysis Using HPLC

**Figure 2:**
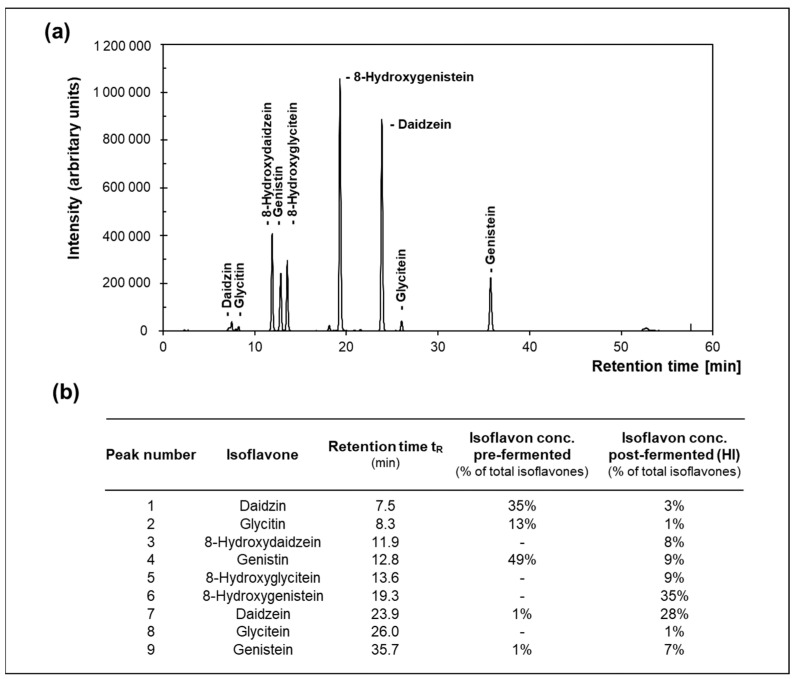
(**a**) Representative HPLC chromatogram of hydroxy-isoflavones (HI)-enriched post-fermented soybean extract at 254 nm and (**b**) the corresponding relative concentrations of identified isoflavones in pre- and post-fermented soybean extract.

Soy isoflavone extract was supplied by Toyo Hakko Co., Ltd. (Aichi, Japan). To obtain an extract enriched in HI, soy isoflavone extracts (pre-fermented samples) were sterilized at 121 °C for 20 min and, subsequently, fermented with *Aspergillus* sp. JCM 22,299 for 8 d at 30 °C with aeration and continuous stirring. After ethanol extraction and two separation steps, the extract was concentrated using evaporation and filtrated through a 30-mesh filter. The obtained post-fermented samples as well as the pre-fermented samples were analyzed in terms of their isoflavone content through HPLC (Prominence UFLC, Shimadzu Corporation, Kyoto, Japan) using a Phenomenex, Kinetex C18 column, 100A (5 μm, 4.6 mm I.D. × 250 mm, Phenomenex Inc., Torrance, CA, USA). Mobile phase A consisted of 0.1% acetic acid and mobile phase B was made of acetonitrile while using a gradient profile (0~50 min (15~35% B), 50~55 min (100% B), 55~60 min (15% B)) at a flow rate of 1.0 mL/min and a temperature of 35 °C. The injection volume was 10 µL and detection was carried out at 254 nm. An external standard curve was applied to calculate the concentration using the peak area (Figure 2). Hence, the concentrations of 8OHGen, 8OHGly and 8OHDai were determined to be 35%, 9% and 8% of total isoflavones content in the post-fermented extract, respectively.

### 2.2. Antioxidant Capacity Assays

The free radical scavenging properties of pre- and post-fermented soy extracts (10 µg/mL) were determined with the ferric-reducing ability of plasma (FRAP) assay and the trolox equivalent antioxidant capacity (TEAC) assay, as previously described [33,34]. For the FRAP assay, which measures how well a test compound reduces ferric ions (Fe^3+^) to ferrous ions (Fe^2+^), pre- or post-fermented soy extracts were added to a 2 mM iron (III) chloride solution with 2,4,6-tris(2-pyridyl)-s-triazine (TPTZ, 1 mM) in acetate buffer (228 mM, pH 3.6). After 15 min of incubation, absorbance was measured at 620 nm in an iEMS reader MF (Labsystems, Helsinki, Finland). Results are given in µmol ascorbic acid equivalents per mg extract. The TEAC assay is related to the reduction of ABTS (2,20-azino-bis-(3-ethylbenzthiazoline-6-sulfonic acid) radical cation by antioxidants. The increase in reduction by pre- or post-fermented extracts was photometrically measured at 750 nm in a Tecan Infinite 200 (Tecan Group Ltd., Crailsheim, Germany) microplate reader and compared to trolox as external standard. TEAC values are given in µmol trolox equivalents per mg extract.

### 2.3. Enzymatic Assays

#### 2.3.1. In Vitro α-Glucosidase Inhibition Assay

A total of 15 µL of diluted (0.05–10 mg/mL) HI extracts was added to 105 μL of 0.1 M phosphate buffer, pH 6.8 and 15 μL of 0.5 U/mL baker yeast α-glucosidase (Sigma-Aldrich, Taufkirchen, Germany). Acarbose was used as a reference inhibitor. After 5 min of pre-incubation at 37 °C, 15 μL of the substrate p-nitrophenyl-α-D-glucopyranoside (10 mM, Sigma-Aldrich, Taufkirchen, Germany) was added and incubated for 20 min at 37 °C in a 96-well microtest plate (VWR, Darmstadt, Germany). The reaction was stopped by adding 50 μL 2 M Na_2_CO_3_ (VWR, Darmstadt, Germany) and the absorbance of samples was measured photometrically at 405 nm (iEMS Reader MF).

#### 2.3.2. In Vitro α-Amylase Inhibition (Disc) Assay

Four filter discs with a diameter of 0.5 cm were placed in a 92 × 16 mm Petri dish (Sarstedt, Nuernbrecht, Germany) filled with medium comprising 1% agar–agar (Carl Roth GmbH & Co. KG, Karlsruhe, Germany) and 1% starch (VWR, Darmstadt, Germany). Then, 80 µL of HI extract at concentrations of 0–10 mg/mL was mixed with 20 µL α-amylase (derived from porcine pancreas, Sigma-Aldrich, Taufkirchen, Germany). Acarbose was utilized as a reference inhibitor. A total of 20 μL of each sample was pipetted onto filter discs and left at 37 °C overnight. After removing the filter discs, plates were incubated with 5 mM iodine in 3% potassium iodide solution (Merck, Darmstadt, Germany). After 15 min, the diameters of the cleared zones were evaluated and the percentage inhibition of α-amylase was calculated. The disc assay was performed on two independent testing days.

#### 2.3.3. In Vitro Dipeptidyl Peptidase-4 (DPP4) Inhibition Assay

The DPP4 inhibitor activities of HI extracts were determined using the DPP4 inhibitor screening kit according to the manufacturer’s instructions (MAK203, Sigma-Aldrich, Taufkirchen, Germany). The HI extract was dissolved in DMSO to a concentration of 100 mg/mL and further diluted to a final concentration of 1 mg/mL, 250 µg/mL and 100 µg/mL with assay buffer. Then, 18 nM of the established DPP4 inhibitor sitagliptin (representing its IC_50_ concentration) served as the positive inhibitor control, whereas assay buffer only was used as the control for DPP4 enzyme activity and was set to 100%. Subsequently, 49 μL assay buffer and 1 μL DPP4 enzyme were mixed with 25 μL of HI extract and 18 nM sitagliptin or assay buffer. After 10 min pre-incubation at 37 °C, a reaction mix of 23 μL assay buffer and 2 μL substrate was given to each well. The fluorescence signal (excitation: 360 nm, emission: 465 nm) was measured in black 96-well microtiter plates at 37 °C over a period of 30 min in 1 min intervals (Tecan Infinite 200 microplate reader).

### 2.4. Testing for Mycoplasma Contamination

All cell lines were regularly tested for mycoplasma contamination via the Mycoplasma Detection Kit for conventional PCR (Venor^®^GeM Classic, Minerva Biolabs, Berlin, Germany) using MB Taq Polymerase (5 Unit/µL, 50 Units). All tested cell lines were found to be mycoplasma-negative.

### 2.5. Sodium-Dependent Glucose Transporter 1 (SGLT1) Assay Using Ussing Chambers in Caco-2/PD7 Cells

SGLT1 was determined in Caco-2/PD7 cells by employing the Ussing chambers methodology [32]. Cells were provided by Edith Brot-Laroche, Unité de Recherches sur la Différenciation Cellulaire Intestinale (Villejuif Cedex, France) and seeded at a density of 1 × 10^6^ cells/well into 6-well Corning^®^ Costar^®^ Snapwell cell culture inserts (0.4 μm pore size, 1.12 cm^2^surface area, Merck, Darmstadt, Germany). Following 21 days of culturing, 0.5 mL of the cell-containing medium was given to the apical side (upper compartment) and 2.5 mL of cell-free medium was seeded into the basolateral side (lower compartment). After 7 days, the apical medium was withdrawn FBS. Only monolayers with a transepithelial electrical resistance (TEER) value exceeding 300 Ω cm^2^, measured via a Millicell ERS-2 Volt-Ohm Meter, equipped with a STX01 planar electrode (Merck, Darmstadt, Germany), were regarded as functional barriers and used in the transport studies. Before starting the experiments, Hank’s balanced salt solution (HBSS, pH 7.2) was heated to 37 °C and oxygenated using an influx of carbogen-gas (95% oxygen, 5% carbon dioxide). HBSS was used to fill half-chambers and wash Caco-2/PD7 monolayers before mounting the Snapwell inserts in Ussing chamber slides. Subsequently, both half-chambers were replenished with HBSS solution containing mannitol (10 mmol/L) apically and glucose (10 mmol/L) basolaterally. The measurement of the transepithelial potential difference was performed at 37 °C under open-circuit conditions using a DVC 1000 amplifier (WPI) and continuous carbogen bubbling. The potential difference was continuously monitored and recorded through Ag–AgCl electrodes and KBR agarose bridges. The short-circuit current (I_SC_; μA cm^−2^) was measured via an automatic VCCMC8 MultiChannel Voltage Current Clamp (Physiologic Instruments) and data were stored using the Acquire & Analyze Data II acquisition software (Physiologic Instruments). The potential difference was allowed to stabilize for 20 min. Then, 10 mM glucose solution was given apically and 10 mM mannitol solution basolaterally. The glucose-stimulated I_SC_ was challenged by applying either pre-fermented extract (1 mg/mL), post-fermented HI-rich extract (1 mg/mL) or phlorizin (0.1 mM) as a positive control for inhibition of SGLT1 activity. The decline in the glucose-induced I_SC_ was assessed.

### 2.6. Induction of CRP in Hep 3B Cells and Measurement of CRP mRNA and Secreted Protein Level

Induction of CRP in Hep 3B cells and measurement of CRP mRNA and secreted protein levels were conducted according to [35,36]. Hep 3B cells were kindly gifted by Claudia Geismann (Laboratory of Molecular Gastroenterology & Hepatology, Department of Internal Medicine I, UKSH-Campus Kiel, 24,105 Kiel, Germany). Cells were cultivated for 5 days in MEM with Earle’s balanced salt solution (EBSS), L-glutamine (PAN Biotech, Aidenbach, Germany) and 2.2 g/L NaHCO_3_; and supplemented with 10% (vol/vol) heat inactivated fetal bovine serum (Gibco™ by Thermo Fisher Scientific GmbH, Life Technologies™, Darmstadt, Germany) and 1% penicillin/streptomycin (PAN Biotech, Aidenbach, Germany). For CRP induction, Hep 3B cells were incubated with 10 µg/mL isoflavone extract in DMSO in serum-free media containing 1 µM of dexamethasone (Dex) and stimulated with interleukin-1ß (IL1ß, 400 U/mL) and interleukin-6 (IL6, 200 U/mL) (both from ImmunoTools GmbH, Friesoythe, Germany) for 18 h for mRNA isolation or 48 h for ELISA analyses. DMSO served as the solvent control at a final dilution of 0.1%.

RNA isolation and quantitative RT-PCR were performed as previously described [37]. In brief, cells were harvested and RNA isolated with peqGOLD TriFast (VWR International, Radnor, PA, USA). The RNA isolation procedure is based on phenol and guanidinium thiocyanate extraction and on separation of RNA, protein and DNA into three phases upon centrifugation after adding chloroform. RNA concentrations and purity (260/280 nm) were determined with a Nanodrop 2000 (Thermo Fisher Scientific GmbH, Life Technologies, Darmstadt, Germany). Gene expression was determined using quantitative RT-PCR with the SensiFAST™ SYBR^®^ No-ROX One-Step Kit (Bioline, Luckenwalde, Germany) via Rotorgene 6000 cycler (Corbett Life Science, Sydney, Australia). Gene expression levels were analyzed using a standard curve and normalized to the expression level of GAPDH. Primers were as follows: CRP forward primer: 5′-CCCTGAACTTTCAGCCGAATACA-3′; CRP reverse primer 5′-CGTCCTGCTGCCAGTGATACA-3′; GAPDH forward primer: 5′-CAATGACCCCTTCATTGACC-3′; and GAPDH reverse primer: 5′-GATCTCGCTCCTGGAAGATG-3′.

To determine the secreted CRP protein levels, the cell culture medium was diluted 1:250 before being used for the ELISA according to the manufacturer’s instructions (Hu-man C-reactive Protein ELISA Kit, Sigma-Aldrich, Taufkirchen, Germany).

### 2.7. Drosophila Melanogaster Feeding Assay Using a High-Starch Diet

The *D. melanogaster* wild-type strain *w^1118^* (#5905, Bloomington Drosophila Stock Center, Indiana University, Bloomington, United States) was maintained under standard conditions in climate cabinets HPP750 or HPP110 (Memmert, Schwabach, Germany) at 25 °C, 60% humidity, and a 12/12 h light/dark cycle [32]. The fruit flies were cultured on Caltech medium (6.0% cornmeal, 5.5% dextrose, 3.0% sucrose, 2.5% inactive dry yeast, 1.0% agar Type II, Kisker, Steinfurt, Germany). Propionic acid (0.3%, Carl Roth, Karlsruhe, Germany) and Tegosept (0.15%, Genesee Scientific, San Diego, SC, USA) were added to the medium as preservatives. The feeding assay was started by transferring freshly eclosed adult animals to CT medium for mating. On day 3, female fruit flies were sorted and transferred onto a starch-based control diet (20% soluble starch (Carl Roth), 5% yeast, 2% agar, 0.18% nipagin, 0.3% propionic acid) or experimental diets that were supplemented with 0.8%, 1.6% or 2.4% of the post-fermented HI-rich extract. A medium containing 1.8 μg/mL acarbose was used as positive control [32]. The mated female flies were then transferred to the respective fresh experimental medium every other day. On day 10, the animals were harvested and ten flies per vial were homogenized for 10 min at 4 °C and 25 Hz in 0.05% Triton X100-containing PBS using a tissue lyser (Qiagen TissueLyser II, Hilden, Germany). The protein and triglyceride content of the fly lysates were measured using a Pierce BCA Protein Assay Kit (Pierce Biotechnology, Rockford, IL, USA) and colorimetric assay reagent (GPO-PAP Kit, Dialab, Wiener Neudorf, Austria), respectively.

### 2.8. Statistics

Statistical analyses were performed using the software GraphPad Prism (Ver. 7.05). The IC_50_ value of glucosidase inhibition by the post-fermented HI-rich extract was calculated using nonlinear regression. Prior to statistical tests, normal distribution of data was approved using the Shapiro–Wilk normality test. An analysis of variance (ANOVA) was conducted for α-amylase inhibition and in vivo fly data, followed by a post-hoc multiple comparison test of Dunnett to compare means of treatment with the post-fermented HI extract to the controls. Results from Ussing chamber experiments, CRP, FRAP and TEAC measurements were analyzed with two-sided unpaired Student’s *t*-tests. In cases without normally distributed data, non-parametric tests were applied. Data from the DPP4 inhibiting assay were tested using the Kruskal–Wallis test and the Dunn’s multiple comparison test. For secreted CRP protein level, the Mann–Whitney test was conducted. *p*-values less than 0.05 were considered significantly different.

## 3. Results

### 3.1. Post-Fermented Hydroxy-Isoflavone (HI)-Rich Soybean Extract Exhibited Significant Inhibitory Activity towards α-Glucosidase and DPP4 In Vitro, but Not towards α-Amylase

**Figure 3:**
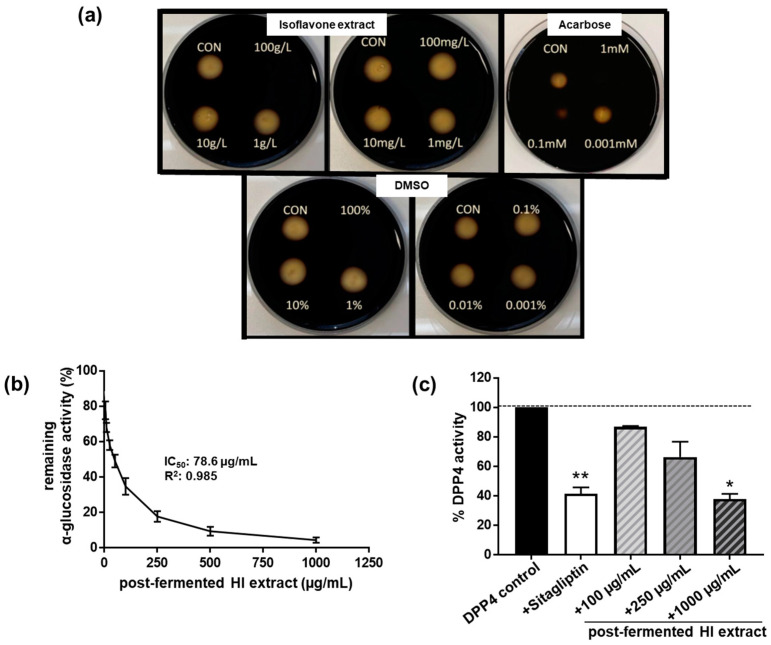
The post-fermented HI-rich extract did not affect (**a**) in vitro α-amylase activity, but inhibited (**b**) α-glucosidase activity as well as (**c**) dipeptidyl peptidase-4 (DPP4) enzyme activity in a dose-dependent manner. For measuring in vitro α-amylase inhibition, the disc diffusion assay was applied. Porcine pancreatic α-amylase was mixed with increasing concentrations of HI extract (final concentrations as indicated) and administered onto the filter discs filled with medium comprising 1% agar–agar and 1% starch. After overnight incubation at 37 °C, starch/agar plates were iodide-stained. α-Amylase inhibition in samples was determined by matching the diameter of the cleared zones. HI extract-, DMSO- (solvent control for the HI extract) and acarbose- (positive control) treated filter discs were compared to control filter discs (CON, α-amylase alone). The assay was conducted on two independent testing days. Exemplary results are shown. α-Glucosidase activity was determined spectrophotometrically. IC_50_ was calculated using GraphPad Prism. DPP4 assay was performed in the presence of the following substances: DPP4 control: assay buffer; sitagliptin: 18 nM; post-fermented HI-rich extract as indicated. Values of remaining DPP4 enzyme activity (in %) compared to the DPP4 control are displayed. Results are mean values of *n* = 2–4 independent experiments. Error bars indicate standard deviation. ** *p* < 0.01; * *p* < 0.05, Kruskal–Wallis test (*p* < 0.001), followed by Dunn’s multiple comparison test, compared to DPP4 control enzyme activity.

In order to test the ability of HI extracts to modulate carbohydrate-hydrolyzing enzymes in vitro, we first examined the influence on α-amylase activity. However, we did not observe a significant modulation of α-amylase enzyme activity by the post-fermented HI-rich extract up to a concentration of 10 mg/mL (Figure 3a).

When we looked at the in vitro inhibition of α- glucosidase activity, we discovered a concentration-dependent inhibitory effect of the soy HI extract (Figure 3b). The IC_50_ value of the extract was estimated to be 78.6 μg/mL (R^2^: 0.985; 95% confidence interval: 60.6–102 µg/mL). The soy HI extract was six times more potent than the positive control acarbose (IC_50_ = 493 µg/mL, R^2^: 0.973; 95% confidence interval: 348–697 µg/mL) at inhibiting α-glucosidase activity.

Furthermore, the HI extract inhibited the dipeptidyl peptidase activity of DPP4 in a dose-dependent manner (ANOVA: *p* < 0.001), leading to an approximately 60% inhibition of enzyme activity at the highest concentration of 1 mg/mL when compared to controls (Figure 3c). A similar inhibition of DPP4 activity was achieved by the inhibitor control sitagliptin, but at a much lower concentration of 18 nM (this equates to 7.33 ng/mL). Thus, HI extract might serve only as a moderate inhibitor of DPP4 enzyme activity.

### 3.2. Pre- and Post-Fermented Soy Isoflavone Extracts Were Moderate Inhibitors of SGLT1-Mediated Glucose Transport

**Figure 4:**
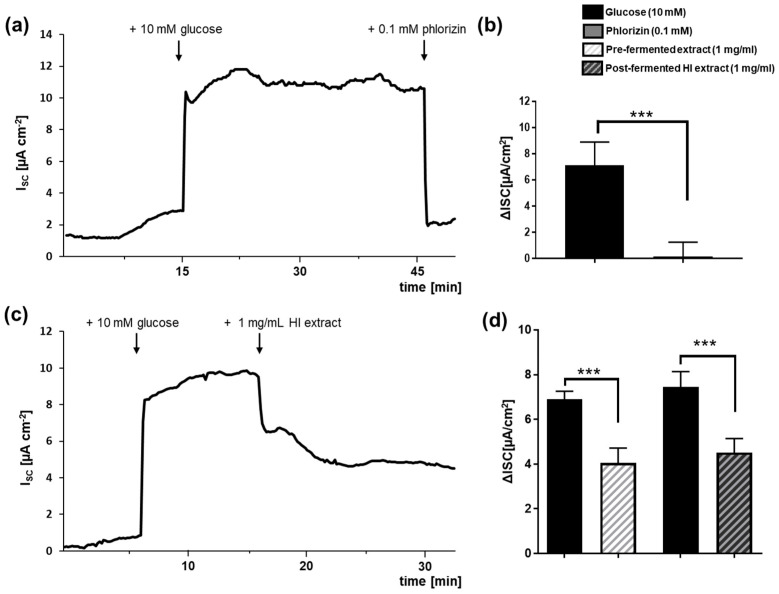
Influence of pre-fermented and HI-enriched post-fermented soy isoflavone extracts on sodium-dependent glucose transporter 1 (SGLT1). SGLT1-dependent glucose transport was measured in Caco-2/PD7 cell monolayers via Ussing chambers. Short-circuit current (I_SC_) was followed over time (exemplary runs depicted in (**a**,**c**)). After addition of 10 mM glucose to the apical side at the indicated time points, the I_SC_ reached a stable plateau within approximately 10 min, before 0.1 mM phlorizin (**a**) as positive control or 1 mg/mL soy isoflavone extract (**c**) was added. The corresponding I_SC_ values are shown in (**b**,**d**) and the I_SC_ values for the pre-fermented extract are shown in (**d**). Error bars indicate standard deviation of *n* = 4–7 independent experiments. *** *p* < 0.001, unpaired t-test. HI: Hydroxy-isoflavone extract.

To examine whether soy isoflavone extracts before (pre-) fermentation and HI-enriched after (post-) fermentation affect SGLT1-mediated glucose transport, we employed Ussing chamber experiments using the Caco-2/PD7 cell monolayer model. Representative runs are given in Figure 4a, c. Adding either pre- or post-fermented extract at a concentration of 1 mg/mL to the Ussing chamber system substantially lowered the glucose-induced short-circuit current from 6.90 ± 0.36 to 4.05 ± 0.66 µA/cm^2^ (pre-fermented extract) and from 7.45 ± 0.69 to 4.51 ± 0.63 µA/cm^2^ (post-fermented HI-rich extract), respectively (Figure 4d). This represents a SGLT1 inhibition of approximately 60% for both extracts. In comparison, glucose uptake was almost completely blocked by the established SGLT1 inhibitor phlorizin at a concentration of 0.1 mM (Figure 4b).

### 3.3. Expression of C-Reactive Protein (CRP)-Coding mRNA and CRP Protein Secretion Were Reduced in Hep 3B Cells by Pre- and HI-Enriched Post-Fermented Soy Extract

**Figure 5:**
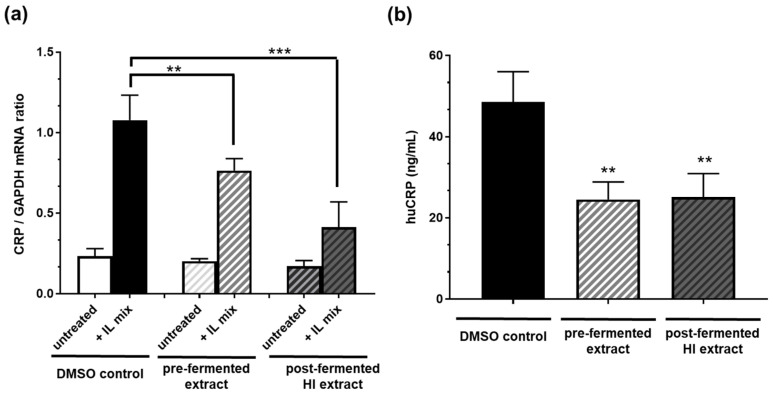
Soy isoflavone extracts before (pre-) and after (post-) fermentation (HI) reduced (**a**) CRP mRNA expression and (**b**) the amount of secreted CRP protein in human hepatoma (Hep B3) cells. Cells were incubated with either pre- or post-fermented extract (10 µg/mL) solved in DMSO. CRP expression was stimulated with 400 U/mL interleukin-1ß and 200 U/mL interleukin-6 (IL Mix). DMSO only served as solvent control. *n* = 6 independent experiments. Results are mean values ± SD. Error bars indicate standard deviation. *** *p* < 0.001; ** *p* < 0.01: unpaired t-test (mRNA) or Mann–Whitney test (secreted protein level) between treatment compared to DMSO solvent control after stimulation.

Incubation of Hep 3B cells with 10 µg/mL pre-fermented soy extract significantly inhibited the mRNA expression of the inflammatory marker CRP after IL1ß plus IL6 stimulation by ca. 30% (Figure 5). An even more potent inhibition of ca. 60% was observed by incubating the cells with post-fermented HI-rich extract (10 µg/mL). However, when analyzing the impact on the level of secreted protein, both pre- and post-fermented extracts significantly reduced the CRP concentration similarly by about 50%.

### 3.4. Post-Fermented HI-Rich Soy Extract Exhibited Higher Antioxidative Capacity Than Pre-Fermented Soy Extract

**Figure 6:**
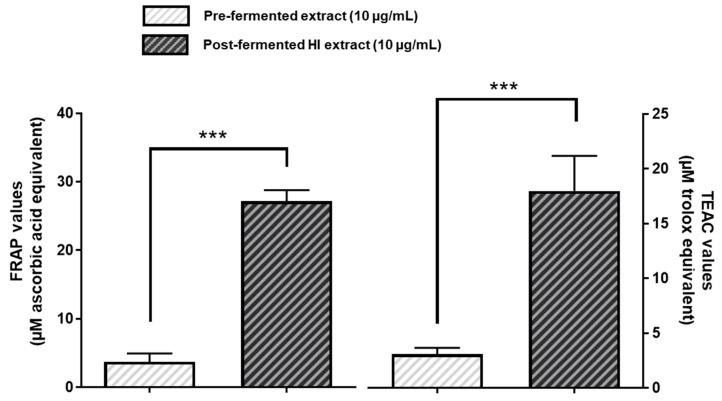
Post-fermented HI-rich soy extract exhibited higher antioxidative capacity than pre-fermented soy extract. For the ferric-reducing ability of plasma (FRAP) assay, pre- and post-fermented soy extracts were incubated with 2 mM iron (III) chloride in the presence of TPTZ for 15 min. The resulting complex between TPTZ and reduced iron (II) was measured photometrically at 620 nm. Results are given in µM ascorbic acid equivalents. The ability to reduce ABTS by pre- or post-fermented soy extracts was investigated in the Trolox equivalent antioxidant capacity (TEAC) assay and measured spectrophotometrically at 750 nm. TEAC values are given in µM trolox equivalents. Results are mean values ± SD of *n* = 6–10 independent experiments. Error bars indicate standard deviation. *** *p* < 0.001: unpaired *t*-test between pre- and post-fermented HI extract.

We next tested the free radical scavenging properties of pre- or post-fermented soy extracts (10 µg/mL) by employing the ferric-reducing ability of plasma (FRAP) assay as well as the Trolox equivalent antioxidant capacity (TEAC) assay, respectively. As shown in Figure 6, in both cases the fermented soy extract with the increased HI content exhibited a significantly higher antioxidative capacity than the pre-fermented soy extract.

### 3.5. Supplementation of a High-Starch Drosophila Melanogaster Diet with Post-Fermented HI-Rich Extract Decreased the Triacylglyceride (TAG) Content of Female Fruit Flies

**Figure 7:**
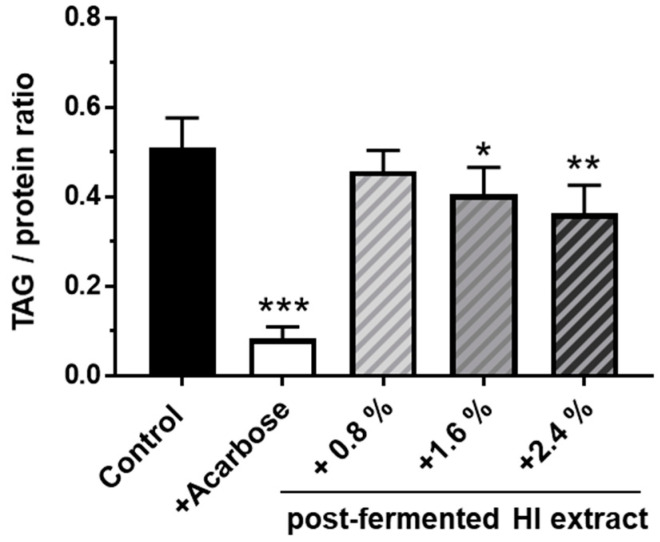
Supplementation of post-fermented HI-rich soy extract decreased the triacylglyceride (TAG) content in *D. melanogaster* fed a high-starch diet. Freshly eclosed male and female fruit flies were mated for 2 days. On day 3, females were allocated to control medium (20% starch, 5% yeast extract) or different experimental diets supplemented with the indicated amount of post-fermented HI-rich soy extract. A diet containing 1.8 mg/L acarbose was used as positive control. After 1 week, the flies were harvested and the TAG to protein ratios were determined. Bars represent the mean values ± SD of n = 6 independent experiments. Error bars show standard deviation * *p* < 0.05, ** *p* < 0.01, and *** *p* < 0.001, ANOVA (*p* < 0.001) with post-hoc multiple comparison test of Dunnett, compared to controls. HI: Hydroxy-isoflavone.

Dietary supplementation of the 20% starch-based diet with increasing concentrations of the post-fermented HI-rich extract led to a dose-dependent reduction of the triglyceride content in 10-day-old female flies (Figure 7). In flies fed 2.4% of the post-fermented HI-rich extract, the TAG to protein ratio was found to be 0.36 compared to control animals which had a value of 0.51. The treatment with the positive control acarbose induced an even more drastic decline in lipid storage to a TAG to protein ratio of 0.08.

## 4. Discussion

The prevalence of T2DM is growing globally; hence, controlling postprandial hyperglycemia and inflammation is central for halting disease progression. Soy isoflavones are believed to play a role in diabetes prevention [38]. The dietary intake of soy products has consistently been inversely associated with the risk of T2DM among women [39]. However, the underlying mechanisms and, in particular, the role of soy-derived HI in diabetes prevention remain unclear. By applying a portfolio of numerous in vitro assays related to various important steps within the glucose metabolism (from intestinal digestion to glucose uptake), as well as assessing potential anti-inflammatory properties, we have addressed this research question in the present study. Furthermore, we have included adequate positive controls (e.g., acarbose, sitaglibtin, phlorizin) in the respective assays.

We have shown that a HI-enriched soy extract demonstrated inhibitory activity towards α-glucosidase, moderately reduced the DPP4 enzyme activity and significantly inhibited SGLT1-dependent glucose transport. Furthermore, the fermented HI-rich extract substantially decreased CRP mRNA and secreted protein levels in cultured Hep B3 hepatocytes. Thus, the HI-rich soy extract mediated antidiabetic properties by addressing multiple targets. Since we observed an inhibition of α-glucosidase but not α-amylase, HI may, nevertheless, exhibit a certain specificity as far as carbohydrate digesting enzymes are concerned. A shortcoming of our present experimental approach may be that we studied only the soy isoflavone-rich extracts (although analytically well characterized) but not their purified constituents, which should be taken into consideration in future studies.

A decrease in intestinal glucose uptake could be an important mechanism in counteracting hyperglycemia [40]. Interestingly, we observed a significant inhibition of SGLT1 due to a HI-rich soy extract, as previously reported for other extracts rich in secondary plant metabolites [32,41]. We did not investigate whether the decrease in glucose uptake was mediated via a competitive inhibition of SGLT1. Phlorizin was used as a positive control in our Ussing chamber experiments. Thus, it would also be interesting to study whether there is a synergistic interaction between phlorizin and isoflavones/HI in terms of SGLT1 inhibition. Furthermore, other glucose transporters, such as Glut4, as a potential target of flavonoids [42] could be considered in response to the treatment with HI in additional studies. In terms of the SGLT1 assay, the fermented isoflavones did not show higher bioactivity than the unfermented extract as far as sodium-dependent glucose uptake was concerned. However, regarding anti-inflammatory properties, we observed a stronger inhibition of CRP gene expression in interleukin 1ß- and interleukin 6-stimulated hepatocytes in response to fermented versus unfermented isoflavones, whereas both extracts reduced the amount of secreted CRP protein to the same extent. Thus, fermentation may affect bioactivity in some but not all assays. Furthermore, it was unclear whether the inhibition of CRP gene expression was via a nuclear factor kappa B-controlled signal transduction pathway, as previously reported for the flavone quercetin in cultured hepatocytes [43]. We further observed a moderate inhibition of DPP4 activity due to HI in vitro. Our data were in line with previous studies indicating that prenyl isoflavones improve glucose homeostasis by inhibiting DPP4 in hyperglycemic rats in vivo [44]. Accordingly, genistein has been shown to inhibit DPP4 in diabetic laboratory mice. This bioactivity was accompanied by an enhanced GLP1 concentration [45], which was not monitored in the present study.

However, we have validated the antidiabetic activity of the fermented HI-rich extract in a starch-based high-sugar diet model of *D. melanogaster*. High-sugar diets have been frequently demonstrated to lead to enhanced triglyceride levels in fruit flies [46,47,48]. By choosing starch as the sole carbohydrate source, we addressed all steps of the carbohydrate degradation pathway including the intestinal enzymes α-amylase and α-glucosidase. Therefore, we cannot currently assess which target molecule(s) is/are responsible for the triglyceride-lowering effect of the post-fermented HI-rich extract. Accordingly, further studies are necessary to clarify the precise in vivo mechanism of action. Overall, data from the present study and literature suggest that structural modifications of isoflavones, either through fermentation or endogenous metabolism, affect their pharmacological properties in terms of their bioactivity and possibly also their bioavailability [16]. The inclusion of additional hydroxyl groups into isoflavone molecules due to fermentation often enhances their bioactivity [23]. In contrast, sulfation [49] or glucuronidation [50], which mainly occur in the small intestine as well as in the liver, are associated with the loss of hydroxyl groups, and thereby decrease the bioactivity of isoflavones. Changes in the bioactivity through structural modifications were also evident in the case of the free radical scavenging activity of the post-fermented HI versus the pre-fermented soy extract. Thus, hydroxylation of isoflavones was accompanied with improved free radical scavenging properties, which has also been reported elsewhere for 8OHGen [25], 3OHDai [51], 6-hydroxyequol [52] and 8OHDai [53]. Accordingly, fermentation of soybean residues with *R. oligosporus* and *L. plantarum* resulted in an improved yield of isoflavone aglycones and gamma amino butyric acid, which led to lowered ROS levels and an increased antioxidative capacity, better blood glucose homeostasis and improved blood biochemistry in STZ-induced hyperglycemic mice [54]. Hence, we cannot fully exclude the possibility that beside HI, other ingredients could have contributed to the antidiabetic and anti-inflammatory effect, seen in our study. Improved free radical scavenging activity due to fermentation could also impact the food quality and shelf life of HI-rich soy derived food. Several efforts have been made to increase the bioavailability of isoflavones from soy beans, including the functional cloning of a soy isoflavone conjugate hydrolyzing β-glucosidase as a potential candidate for soy isoflavone bioavailability enhancement [16]. Although the bioavailability of genistein and daidzein has been studied in laboratory rodents [55,56], as well as in humans [57,58], little is known in terms of the bioavailability (e.g., plasma and tissue concentration) of HI including 8OHGen and 8OHGly. Nevertheless, it has been suggested that 8-OHDai is relatively easily absorbed in rats and distributed to peripheral tissues [59]. Such studies are necessary to evaluate whether the isoflavone concentrations used in in vitro studies are physiologically achievable under in vivo conditions. On the other hand, bioavailability was not an issue when isoflavones and HI inhibited intestinal targets, such as α-glucosidase and SGLT1,96 identified here.

## 5. Conclusions

A soy isoflavone extract rich in 8-hydroxygenistein, 8-hydroxyglycitein and 8-hydroxydaidzein exhibited antidiabetic properties in vitro and in an in vivo diabetes model of *Drosophila melanogaster*. Such an extract may have the capability to serve as a dietary natural plant bioactive for prevention strategies in terms of T2DM. However, in the future, the potential antidiabetic and anti-inflammatory properties of HI need to be validated in laboratory rodents, as well as in human intervention studies, also taking their bioavailability into account.

## Data Availability

The data presented in this study are available upon reasonable request from the corresponding author.
